# Comparative Mitogenomic Analysis of the *Eurydema* Genus in the Context of Representative Pentatomidae (Hemiptera: Heteroptera) Taxa

**DOI:** 10.1093/jisesa/iez122

**Published:** 2019-12-16

**Authors:** Wanqing Zhao, Qing Zhao, Min Li, Jiufeng Wei, Xianhong Zhang, Hufang Zhang

**Affiliations:** 1 Department of Biology, Xinzhou Teachers University, Xinzhou, China; 2 Department of Entomology, Shanxi Agricultural University, Taigu, China; 3 Department of Biology, Taiyuan Normal University, Taiyuan, China

**Keywords:** Pentatomidae, *Eurydema*, mitogenome, phylogenetic relationship

## Abstract

The family Pentatomidae, the largest within the superfamily Pentatomoidae, comprises about 5,000 species; many of which are economically important pests. Although the phylogeny of Pentatomidae species has been studied using various molecular markers, their phylogenetic relationships remain controversial. Recently, mitochondrial genomes (mitogenomes) have been extensively employed to examine the phylogenetics and evolution of different insects, and in this study, we sequenced complete/near-complete mitochondrial genomes from five shield bug species of *Eurydema* to gain a better understanding of phylogenetic relationships in the Pentatomidae. The five mitogenomes ranged in length from 15,500 to 16,752 bp and comprised 13 protein-coding genes (PCGs), 22 transfer RNAs (tRNAs), 2 ribosomal RNAs (rRNAs), and a control region. We compared mitogenomic characteristics of the Pentatomidae and constructed phylogenetic trees using Bayesian inference and maximum likelihood methods. Our results showed that gene arrangements, base composition, start/stop codons, gene overlaps, and RNA structures were conserved within the Pentatomidae and that congeneric species shared more characteristics. Saturation and heterogeneity analyses revealed that our PCGs and PCGRNA datasets were valid for phylogenetic analysis. Phylogenetic analyses showed consistent topologies based on BI and ML methods. These analyses strongly supported that *Eurydema* species belong to the tribe Strachiini, and formed a sister group with Pentatomini. The relationships among *Eurydema* species were shown to be consistent with their morphological features. (Strachiini + Pentatomini) was found to be a stable sibling of the clade comprising Cappaeini, Graphosomini, and Carpocorini. Furthermore, our results indicated that *Graphosoma rubrolineatum* (Heteroptera: Pentatomidae) belongs to the Pentatominae and not the Podopinae.

The Pentatomidae, or stink bugs, are an important group of Heteroptera and the largest family within the superfamily Pentatomoidae (Hemiptera: Heteroptera). Worldwide, this family consists of ~5,000 known species belonging to >800 genera and 10 subfamilies (Wang 2010, Zhao et al. 2018). In China, Pentatomidae species belong to four subfamilies: Asopinae, Pentatominae, Phyllocephalinae, and Podopinae (Rider et al. 2002; Rider and Zheng 2002, 2005). In previous studies, high-level relationships within the Heteroptera have been a primary focus, whereas there has been comparatively limited research conducted on the phylogenetic relationships of subfamilies and tribes. To date, few molecular markers or morphological characteristics have been applied in analyzing the phylogeny of the Pentatomidae, and accordingly, the phylogenetic relationships among subfamilies and tribes remain controversial (Grazia et al. 2008, Yuan et al. 2015). Yang (1962) suggested a classification system of nine tribes, in which *Eurydema* was placed within the Palomenini. However, Rider (2015) subsequently classified *Eurydema* within the Strachiini on the website of Pentatomoidea Home Page (http://www.ndsu.nodak.edu/ndsu/rider/Pentatomoidea/). In addition, on the basis of morphological data, some researchers have considered that the genus *Graphosoma* belongs to the tribe Graphosomini of Pentatominae (Hsiao1977, Liu 2007), whereas others have upgraded it to the Podopinae (Rider 2002, 2015; Xing 2013).

In China, species within the genus *Eurydema* Laporte (Heteroptera: Pentatomidae) are known as agricultural pests of cruciferous plants, with outbreaks occurring from June to August. Whereas some of these *Eurydema* species are widespread across the Palaearctic (e.g., *Eurydema dominulus* and *Eurydema oleracea*), others are endemic (e.g., *Eurydema maracandica* and *Eurydema liturifera*) (Yang 1962, Hsiao 1977, Rider 2006, Henry 2009). Although in general, phenotypic variation within the species of the genus *Eurydema* with respect to color has aided species identification compared with true bugs, it has been of limited utility in distinguishing closely related species. Additionally, multiple intraspecific phenotypes can be found, making precise identification difficult. In order to overcome these taxonomic difficulties, some authors have examined the utility of DNA barcoding and the characteristics of male genitalia (Jung et al. 2011; Akhter et al. 2015; Zhao et al. 2017a,b). However, mitogenome analysis may offer a different perspective with regards to determining the phylogeny and diversification of the genus *Eurydema* and has a high probability of resolving the evolution of this genus (Breeschoten et al. 2016).

Insect mitogenome is double-stranded circular DNA molecules with length of 14–17 kb, and typically contains 13 protein-coding genes (PCGs), 22 transfer RNA genes (tRNAs), and 2 ribosomal RNA genes (rRNAs) (Wolstenholme 1992, Françoso et al. 2016, Yang et al. 2018). Mitogenome harbors a number of key molecular markers that can be used to resolve phylogenetic relationships at multiple taxonomic levels and are characterized by a number of informative features, including, length variation, altered RNA secondary structures, rearranged genes, and hyper variable control regions (Dowton et al. 2002, Cameron et al. 2010, Colinet et al. 2017, Djoumad et al. 2017). Owing to their relatively conserved gene content and organization, rapid evolutionary rate, high genetic information content, and abundant genome-level features, insect mitogenomes have been widely used in phylogenetic analyses, species identification, and evolution at different taxonomic levels (Ma et al. 2012, Hegedusova et al. 2014, Liu 2017, Wu et al. 2019).

At present, 167 complete or near-complete mitogenomes of heteropteran species are available in GenBank, and there have been numerous studies that have used these mitogenomes (Fischer et al. 2000; Weirauch and Schuh 2010; Li et al. 2012, 2017; Wang et al. 2016). However, only 14 mitogenomes have been sequenced for Pentatomidae to date. Therefore, sequencing additional mitogenomes from species within the Pentatomoida is needed.

Herein, we sequenced the mitogenomes of five *Eurydema* species and compared the mitogenome features of the *Eurydema* with those of the 14 previously sequenced Pentatomidae mitogenomes. We analyzed the A+T content and AT-skew of different partitions, codon usage, and nucleotide substitution of 13 PCGs, and the secondary structure of RNA genes. The applicability of two datasets containing the 13 PCGs and all 37 genes for phylogenetic analysis were evaluated by heterogeneity and substitution saturation analysis. Finally, we examined phylogenetic relationships using Bayesian inference (BI) and maximum likelihood (ML) methods to confirm the main tribes of Pentatominae and species within the genus *Eurydema*.

## Materials and Methods

### Taxon Sampling and Mitogenome Sequencing

The specimens used in this study were collected in China between 2013 and 2016, and the detailed information listed in Table 1. All samples were preserved in 100% ethanol and stored at −20°C. Total genomic DNA was extracted from leg muscles using a ONE-4-ALL Genomic DNA Mini-Prep Kit (BS88504; Sangon, Shanghai, China). The mitogenomes were sequenced using the whole-genome shotgun method on an Illumina Miseq platform (Personalbio, Shanghai, China). After filtering low-quality and adapter contaminated reads, A5-miseq version 20150522 (Coil et al. 2015) was used for contig assembly.

**Table 1.** Sequence information included in the present study

Species	Common name	Accession number	Locality	Collection date
*Aradacanthia heissi*	Flat bug	HQ441233	No record in GenBank	No record in GenBank
*Urochela quadrinotata*	Four-spotted shield bug	NC_020144	No record in GenBank	No record in GenBank
*Megacopta cribraria*	Bean plataspid	NC_015342	No record in GenBank	No record in GenBank
*Coptosoma bifaria*	Double-row turtle bug	EU427334	No record in GenBank	No record in GenBank
*Macroscytus gibbulus*	Horizontal leather burrower bug	NC_012457	No record in GenBank	No record in GenBank
*Eusthenes cupreus*	Discolor giant bugs	NC_022449	No record in GenBank	No record in GenBank
*Coridius chinensis*	Melon black bug	JQ739179	No record in GenBank	No record in GenBank
*Halyomorpha halys*	Brown marmorated stink bug	NC_013272	No record in GenBank	No record in GenBank
*Pentatomidae* sp.	Stink bug	KM244699	No record in GenBank	No record in GenBank
*Graphosoma rubrolineatum*	Striped shield bug	NC_033875	No record in GenBank	No record in GenBank
*Rubiconia intermedia*	Bead bug	KP207596	No record in GenBank	No record in GenBank
*Dolycoris baccarum*	Sloe bug	KC460537	No record in GenBank	No record in GenBank
*Nezara viridula*	Southern green stink bug	EF208087	No record in GenBank	No record in GenBank
*Eurydema gebleri*	Cross grain cabbage bug	NC_027489	No record in GenBank	No record in GenBank
*Eurydema maracandica*	Xinjiang cabbage bug	MF135553	No record in GenBank	No record in GenBank
***Eurydema dominulus***	Cabbage bug	MG584833	Linyi, Shandong	2 Aug. 2014
***Eurydema ventralis***	Border-radius cabbage bug	MG584837	Altay, Xinjiang	1 Aug. 2011
***Eurydema qinlingensis***	Qinling cabbage bug	MG584836	Ankang, Shaanxi	17 July 2016
***Eurydema oleracea***	Blue cabbage bug	MG584835	Tarbagatay, Xinjiang	8 Aug. 2016
***Eurydema liturifera***	India-Burma cabbage bug	MG584834	Pu’er, Yunnan	1 Aug. 2006

### Sequence Annotation and Analysis

The five newly sequenced mitogenomes were annotated using Geneious 10.1.3 (Kearse et al. 2012) via comparison with the previously published sequence of the *E. maracandica* mitogenome (Zhao et al. 2017a). Annotations of 13 protein-coding regions were edited manually by predicting open reading frames using the invertebrate mitochondrial code. tRNA genes were identified based on their characteristic cloverleaf secondary structure using the MITOS web server (Bernt et al. 2013). rRNA genes were identified by comparing nucleotide sequences with those of previously reported mitogenomes.

Along with the complete mitogenomes in the present study, 15 complete Heteroptera mitogenomes were downloaded from the NCBI GenBank (Table 1). The mitogenome of *Aradacanthia heissi* (Aradidae) was used as an outgroup. The sequences of 13 PCGs, 22 tRNAs and two rRNAs were extracted from each mitogenome. PCGs were aligned according to codon-based multiple alignments using the MUSCLE algorithm in MEGA 7.0 (Kumar et al. 2016). Genes for RNAs were aligned with MUSCLE using default parameters in MEGA 7.0. Alignments of individual genes were concatenated in SequenceMatrix (Vaidya et al. 2011) according to the following two matrices for phylogenetic analyses: i) the PCGs matrix, including all three codon positions of the 13 PCG genes (13PCG); ii) the PCGRNA matrix, including 13 PCG genes, 22 tRNA genes, and 2 rRNA genes. The codon usage of PCGs and nucleotide composition of different regions were analyzed using MEGA 7.0. The AT- and GC-skew values were calculated as follows: AT skew = (*A-T*)/(*A+T*) and GC skew = (*G-C*)*/*(*G+C*).

### Phylogenetic Analyses

Phylogenetic analyses were conducted using BI. The heterogeneity of sequence divergence within datasets was analyzed using AliGROOVE (Kück et al. 2014). In order to avoid incorrect phylogenetic inferences, Xia’s saturation index (*Iss*) was estimated and compared to the critical values (*Iss.c*) using DAMBE (Xia 2018). BI analysis was performed using MrBayes3.2 (Ronquist et al. 2012) under a GTR + G + I modelselected by jModeltest (Darriba et al. 2012), in which 1,000,000 generations were run, with four simultaneous Markov chains (one cold chain and three heated chains), and trees were sampled after every 1,000 generations. The first 25% of generations were discarded as burn-in when average standard deviation of split frequencies were >0.01. The ML trees were constructed using RAxML (Alexandros 2014) under the GTR+GAMMA model, and the node support values were assessed by bootstrap re-sampling (BP) calculated using 1,000 replicates. Phylogenetic informativeness (PI) of data partitions based on the tree constructed using the combined data of PCGs was measured using PhyDesign (López-Giráldez and Townsend 2011).

## Results

### Genome Organization

We sequenced the complete mitogenomes of *E. dominulus, Eurydema ventralis, E. oleracea*, and *Eurydema qinlingensis*, and the near-complete mitogenome of *E. liturifera*, which was lacking part of the control region. The mitogenome sequences ranged in size from 15,500 bp (*E. ventralis*) to 16,752 bp (*E. dominulus*), whereas the size of the near-complete mitogenome of *E. liturifera* was 15,585 bp. The failure to amplify part of the control region in *E. liturifera* may be due to its high variability and/or extreme length. The five annotated mitogenomes have been deposited in GenBank with accession numbers as per Table 1. The determined sequences are double-stranded circular molecules that in common with most other heteropteran mitogenomes, encode 37 genes (13 PCGs, 22 tRNAs, and 2 rRNAs). The majority of these genes (23) are encoded by the heavy strand (J-strand), with the remaining 14 genes (*trnQ*, *trnC*, *trnY*, *trnF*, *nad5*, *trnH*, *nad4*, *nad4L*, *trnP*, *nad1*, *trnL1(CUN)*, *trnV*, *16S rRNA*, and *12S rRNA*) being located on the light strand (N-strand). The organizations of the five mitogenomes are shown in Supp file (online only).

Although we found little variation in the length of the PCGs, tRNAs and rRNAs of the 14 sequenced Pentatominae mitogenomes, the lengths of control and intergenic regions showed more pronounced variation. Within the Pentatominae mitogenomes, the total length of PCGs, tRNAs, rRNAs, and the control region ranged from 11,004 to 11,027 bp; 1,459 to 1,506 bp; 2,060 to 2,107 bp; and 543 to 2,190 bp; respectively. The Pentatominae mitogenomes have compact arrangements, and both gene overlaps and intergenic spacers were observed, with the former ranging from 1 to 8 bp with a total length of 22–32 bp. The longest overlap was that of the gene pair *trnW*/*nad1* (8 bp), and we found that overlaps for the gene pair *trnC*/*trnW*, *atp8*/*atp6*, and *nad4*/*nad4L* are completely conserved in the *Eurydema* mitogenomes. Intergenic spacers ranged in length from 1 to 31 bp, with a total length of 105–167 bp, among which the spacer between the gene pair *trnS2*/*nad1* (27–29 bp) was found to be the longest intergenic region.

### Nucleotide Composition and Codon Usage

**Fig. 1.**
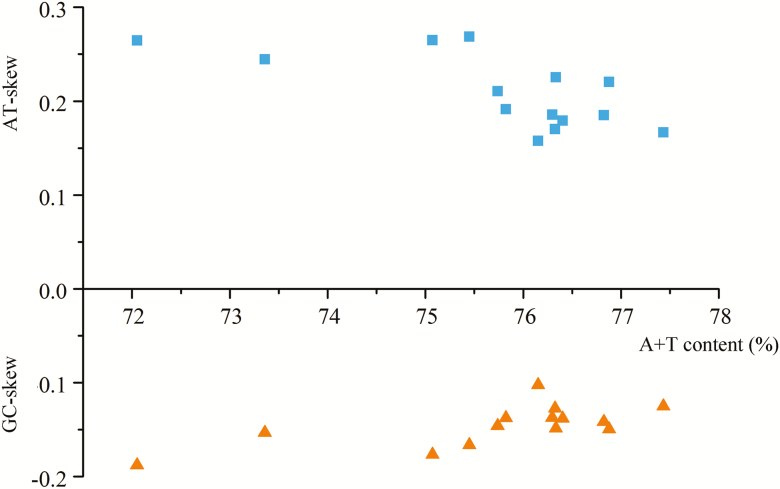
AT skew and GC-skew of Pentatomidae mitogenomes.

**Fig. 2.**
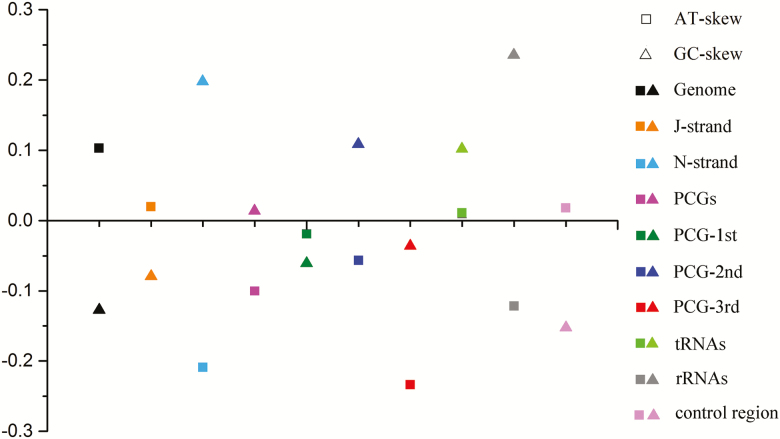
AT skew and GC skew of different databases.

Data relating to the nucleotide composition, whole mitogenome AT- and GC-skew values, J-strands, N-strands, PCGs, PCG-1st, PCG-2nd, PCG-3rd, tRNAs, rRNAs, and control regions of the Pentatominae mitogenomes are presented in Figs. 1 and 2. Overall, the A+T content of these mitogenomes is significantly higher than that of G+C content, and strongly skewed toward A and T. For the three codon positions of protein-coding genes, PCG-3rd (79.9%) has the highest content of A+T, and PCG-1st (72.32%) the lowest. The A+T content of the N-strand is slightly higher than that of the J-strand, with an average A+T content of 75.59%. Among the 13 PCGs, *atp8* has the highest A+T content (83.1%), whereas *cox1* has the lowest (70.3%).

For all sequenced Pentatominae mitogenomes, the AT-skew is greater than 0 (ranging from 0.1578 to 0.2645), whereas the GC-skews are all less than 0 (from −0.1877 to −0.1023). Whole sequences show moderate A/C skew, J-strands show slight A/C skew, N-strands show obvious T/G skew, PCGs show moderate A skew and slight C skew, PCGs-1st shows slight T/G skew, PCGs-2nd shows slight T skew and moderate G skew, PCGs-3rd shows obvious T skew and slight C skew, tRNAs show slight Askew and moderate A skew, and rRNAs show moderate T skew and obvious G skew.

**Fig. 3.**
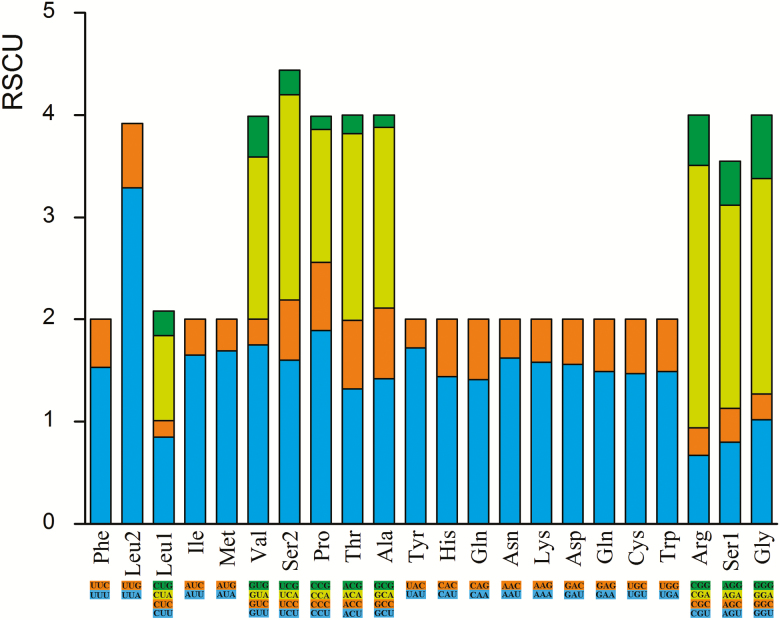
The codon usage of PCGs in Pentatomidae mitogenomes.

The results of statistical analysis of the relative synonymous codon usage (RSCU) in mitogenome PCGs are shown in Fig. 3. The most frequently used codons are UUA (Leu), CGA (Arg), and GGA (Gly), whereas the two most infrequently used codons are GCG (Ala) and CCG (Pro). Synonymous codons ending with an A or U are more prevalent than those ending in a G or C. For instance, UUU (RSCU = 1.53) is more common than UUC (RSCU = 0.47) for Phe, CAU (RSCU = 1.44) is more common than CAC (RSCU = 0.56) for His, and AUU (RSCU = 1.65) is more common than AUC (RSCU = 0.35) for Ile. Although the amino acid compositions of the Pentatominae mitogenome PCGs are similar, we found that their proportions are not the same. Leu was observed to be the most commonly represented amino acid, followed by Phe, Ile, and Met, and therefore, the codons corresponding to these common amino acids also have relatively high proportions.

### Protein Coding Genes

**Fig. 4.**
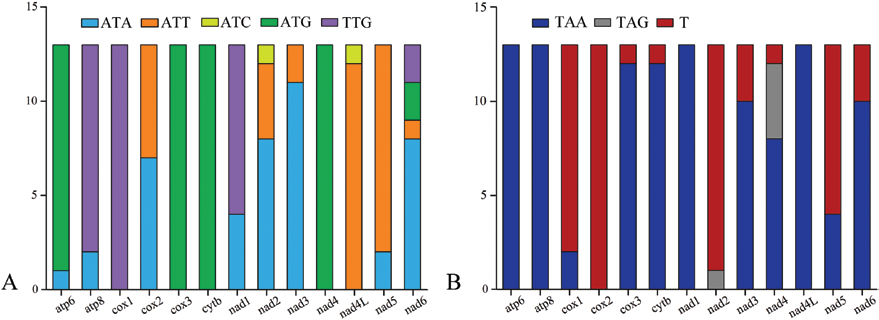
Start (A) and stop (B) codons usage of Pentatomidae protein-coding genes.

**Fig. 5.**
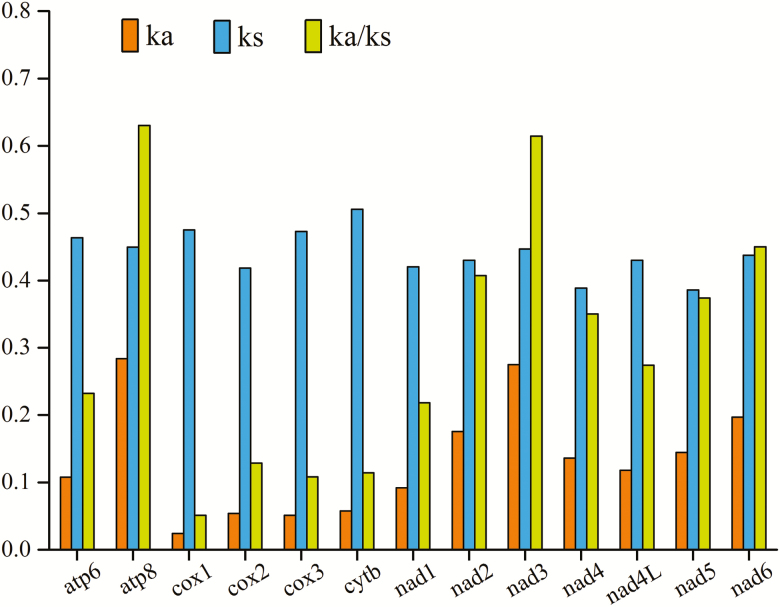
The Ka, Ks, and Ka/Ks values of protein-coding genes.

Among the nucleotide sequences of the PCGs in Pentatominae mitogenome, we found that *cox1* had the highest conserved sites (66.17%), whereas that for *atp8* (27.78%) is the lowest. In terms of amino acid sequences, the proportion of conserved sites in the *cox1* (86.35%) and *cytb* (74.14%) genes was observed to be higher than those in other genes, whereas the proportion in *nad2* (33.03%) was the lowest. Conservation of PCGs is also reflected in the usage of start and stop codons. The results of statistical analysis of Pentatominae mitogenome start/stop codons are shown in Fig. 4. Although most PCGs were found to initiate with a typical start codon (ATN), *atp8*, *cox1*, and *nad1* invariably begin with TTG. Furthermore, all PCGs ended with complete termination codon TAA/TAG, except *cox1*, *cox2*, *nad3*, and *nad5* ended with truncated stop codon (T). Calculation of the rates of nonsynonymous (Ka) and synonymous (Ks) nucleotide substitutions revealed that the Ka/Ks ratios for all 13 PCGs were considerably <1 (<0.63), thereby indicating evolution under purifying selection (Fig. 5). All mitochondrial PCGs can be used to examine phylogenetic relationships. The highest Ka/Ks value was found in *atp8*, whereas *cox1* appears to have the lowest evolutionary rate. *atp8* can be used to analyze intraspecific relationships, whereas the four genes *cox1*, *cox2*, *cox3*, and *cytb* is useful for DNA barcoding.

### Transfer RNA and Ribosomal RNA Genes

**Fig. 6.**
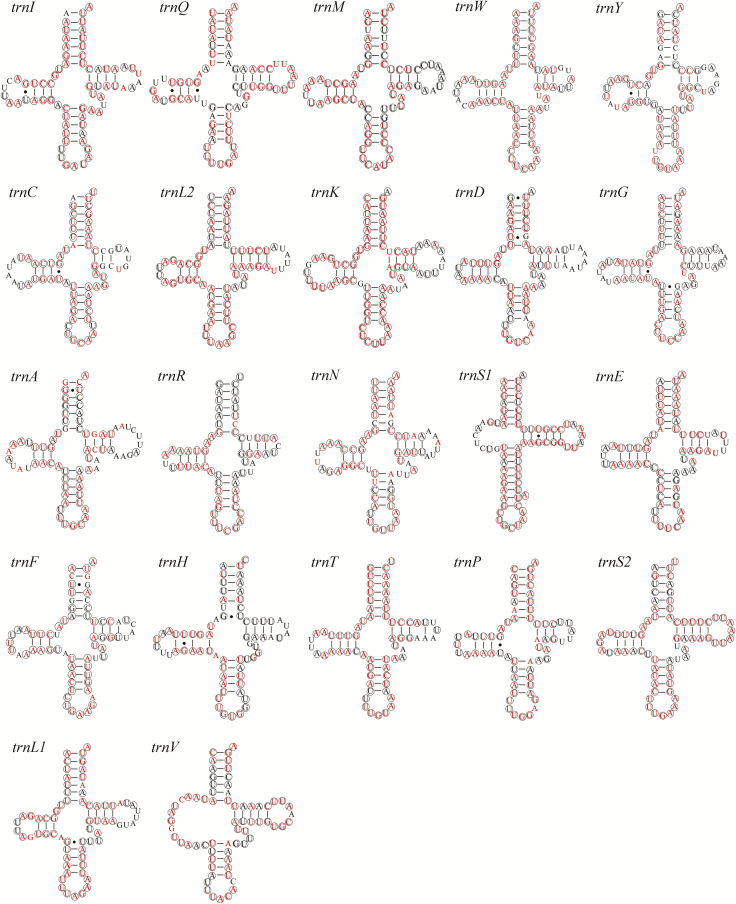
Potential secondary structure of tRNA in *Eurydema dominulus*. The conserved sites within *Eurydema* were labeled with circles, and conserved sites within Pentatominae were marked with red color.

In common with other Pentatominae mitogenomes, we found that the five sequenced *Eurydema* mitogenomes contain 22 tRNA genes, with lengths ranging from 62 to 72 bp. Among these, 20 were predicted to fold into the typical cloverleaf secondary structure, whereas the remaining two tRNAs lack the dihydrouridine (DHU) stem and forma loop. The predicted secondary structures of the 22 tRNAs of *E. dominulus* are shown in Fig. 6. The DHU stem, anticodon stem, TΨC stem, and amino acid acceptor stems of *trnL1(CUN)*, *trnL2(UUR)*, *trnS1(AGN)*, and *trnS2(UCN)* show100% identity within *Eurydema*. Similarly, the anticodon loops in *trnW*, *trnK*, *trnL2*, and *trnF* show 100% identity within the Pentatominae. We detected a total of seven pairs of conserved G-U mismatches within the Pentatominae, which contribute to maintaining the stability of tRNA secondary structures. Additionally, A-G in the DHU stem of *trnH* and C-U in the anticodon stem of *trnM* are also conserved across *Eurydema*.

**Fig. 7.**
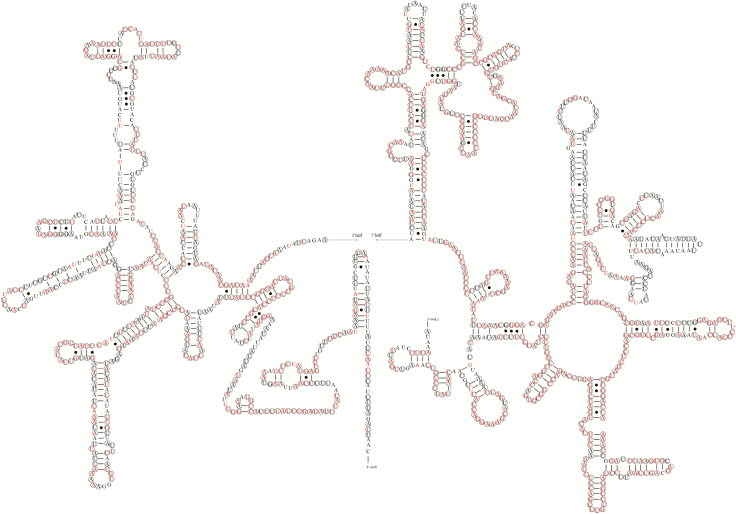
Potential secondary structure of 16S rRNA in *Eurydema dominulus*. The conserved sites within *Eurydema* were labeled with circles, and conserved sites within Pentatominae were marked with red color.

**Fig. 8.**
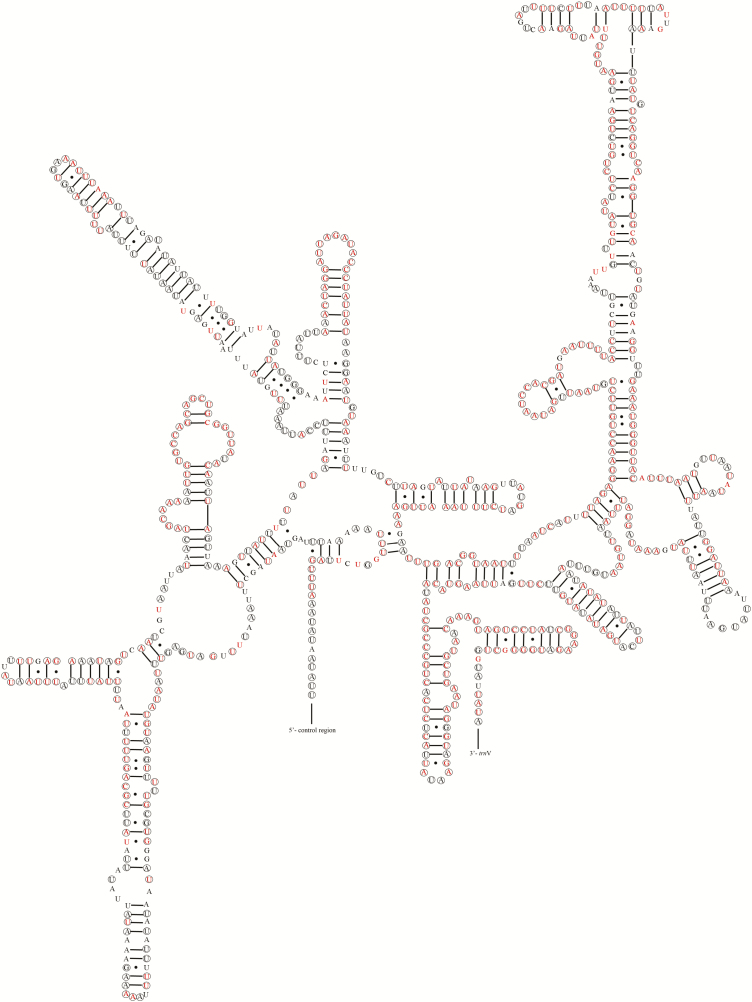
Potential secondary structure of 12S rRNA in *Eurydema dominulus*. The conserved sites within *Eurydema* were labeled with circles, and conserved sites within Pentatominae were marked with red color.

Mitogenomes of the Pentatominaeare characterized by large rRNA (*16S rRNA*), located between *trnL1(CUN)* and *trnV*, and small rRNA (*12S rRNA*) inserted between *trnV* and the control region. The predicted secondary structures of the *E. dominulus* rRNAs are depicted in Figs. 7 and 8. The stem region structure of rRNAs is more highly conserved than the loop structure. The *16S rRNA* contained five domains (the third domain is absent, as in other arthropods) with 44 predicted stems, containing 54.38% conserved sites within the Pentatominae. The *12S rRNA* contains three domains with 26 predicted stems, containing 53.42% conserved sites within the Pentatominae.

### Control Region

Compared with other regions, the control regions of the Pentatominae mitogenomes between the *12S rRNA* and *trnM* exhibited more variation in length, ranging from 543 to 2,190 bp. It is obvious that the length variation among them contribute to the total length differences of their mitogenomes. This region also harbored the highest A + T content (avg. 76.64%) in the mitogenomes, whereas AT skewness and GC skewness were significantly different, which was also observed in other insect mitogenomes from Heteroptera.

**Fig. 9.**
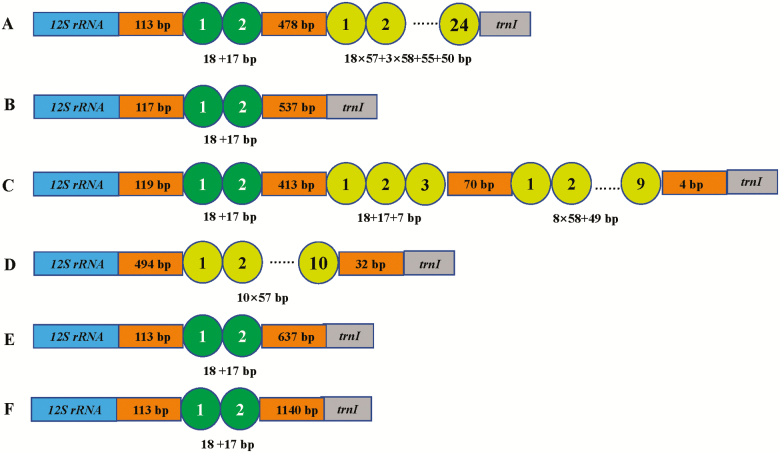
Organization of the control region in *Eurydema* mitochondrial genomes. (A) *Eurydema dominulus*; (B) *Eurydema maracandica*; (C) *Eurydema oleracea*; (D) *Eurydema qinlingensis*; (E) *Eurydema ventralis*; and (F) *Eurydema gebleri*.

The comparison of tandem repeats in control regions of *Eurydema* mitogenomes was shown in Fig. 9. The length and copies of repeat units were different among five species. However, the first repeat unit of 18bp was found in all regions except *E. qinlingensis*.

### Phylogenetic Analysis

**Fig. 10.**
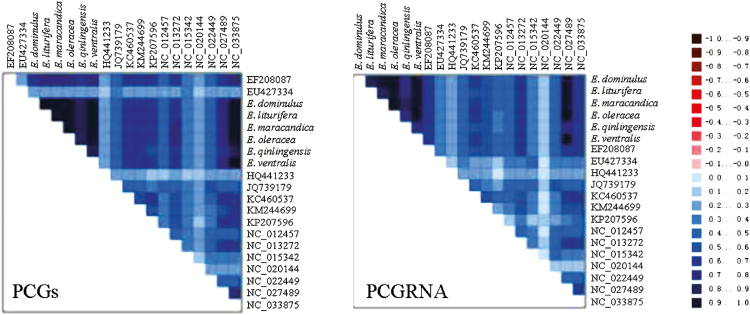
Heterogeneity analysis of PCGs and PCGRNA datasets.

PCGs and PCGRNA datasets were used to construct phylogenetic trees using BI and ML methods. For both datasets, *Iss* values were lower than those of *Iss.c*: PCGs [*Iss*(0.272) <*Iss.c* (0.851)] and PCGRNA [*Iss* (0.516) <*Iss.c* (1.215)]. In order to evaluate sequence variation heterogeneity, the two datasets were analyzed using AliGROOVE, and we found that there was low heterogeneity in the sequence composition of both datasets (Fig. 10). Overall, the saturation and heterogeneity analyses indicated that the two datasets were suitable for further phylogenetic investigation.

**Fig. 11.**
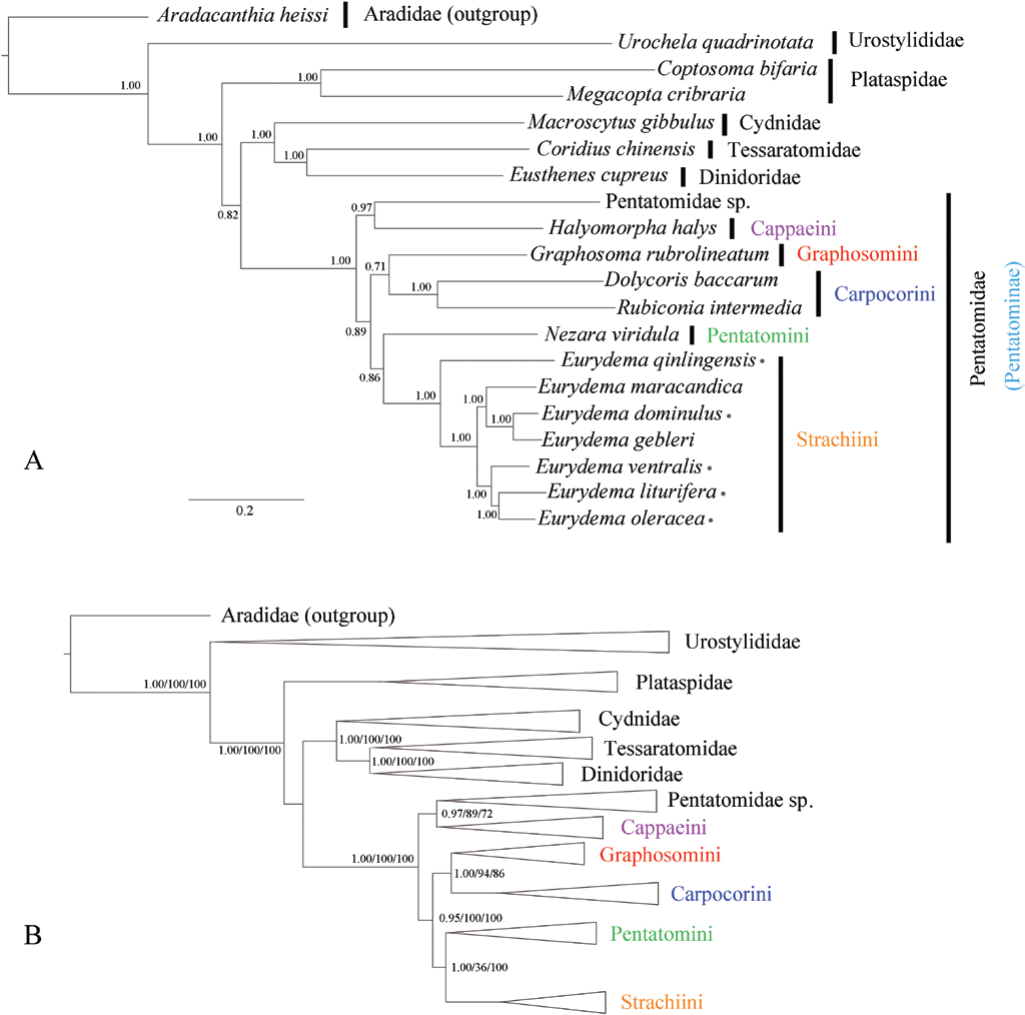
Phylogenetic trees based on Pentatomidae mitogenomes. (A) The topology from the analysis of BI-PCGs. (B) The schematic version of trees from the analysis of BI-PCGRNA, ML-PCGs and ML- PCGRNA.

The final alignments contained 11,609 and 16,579 sites in the PCGs matrix and PCGRNA matrix, respectively. The monophyly of each family and tribe were generally well supported in both BI and ML trees (Fig. 11); most posterior probabilities (PP) are 1.00 and bootstrap pseudoreplicates (BP) are 100. The topology of the Pentatomoidea was as follows: (Urostylididae + (Plataspidae +((Cydnidae +(Dinidoridae +Tessaratomidae)) +Pentatomidae))). The phylogenetic analyses performed in the present study indicated that *Eurydema* species belong to Strachiini, which form a sister group with Pentatomini (PP= 1.00/1.00 and BP = 100/100). *Graphosoma rubrolineatum* was resolved as a sister to Carpocorini, suggesting that this species belongs to the Graphosomini tribe of Pentatominae (PP = 0.71/1.00 and BP = 86/94). The stable clade (Strachiini + Pentatomini) was identified as a sibling to the clade comprising Cappaeini, Graphosomini, and Carpocorini (PP = 1.00/1.00 and BP = 100/100). In addition, *E. qinlingensis* was found to be the first diverging clade within *Eurydema*, whereas the others divided into two clades. *E. ventralis*, *E. liturifera*, and *E. oleracea* formed the clade I, and *E. maracandica*, *E. gebleri*, and *E. dominulus* formed the clade II (PP = 1.00/1.00 and BP = 100/100).

### Phylogenetic Informativeness

**Fig. 12.**
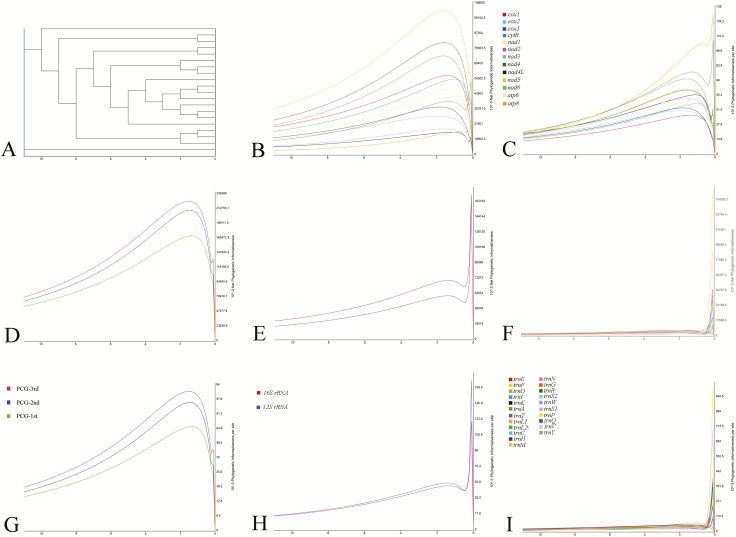
The phylogenetic information of mitochondrial genes/characters.

We calculated the net and per site PI values along the root-to-tip axis for each gene and the three codon positions of PCGs (Fig. 12). PI analysis measures the power of a set of characters to resolve branching order in a phylogenetic tree. The PI curves for PCGs were found to be similar in shape, with a steady increase from the root, to a maximum close to the tip at a hierarchical level that defines the phylogenetic relationships, and then dropped rapidly. For PCGs, *atp8* had the lowest net value (0.77), whereas *nad5* had the highest per site PI value (103.57). The third codon positions had the highest PI along the entire root-to-tip axis both for net and per site values. The PI curves for functional RNAs were similar in shape, with a peak near the tip. For rRNAs, we obtained contrasting net and per site PI results. Specifically, the per site PI of *16S rRNA* (70.09) was higher than that *12S rRNA* (51.93), whereas the net PI of *12S rRNA* (1.43) was slightly higher than that of *16S rRNA* (1.32). For tRNAs, *trnM* and *trnI* had the highest net PI values, whereas *trnS1* had the highest per site PI. The high PI values of PCGs and rRNAs resolved tribe relationships within Pentatominae, whereas tRNAs corresponded to generic relationships.

## Discussion

In this study, we describe the mitochondrial genomes of five *Eurydema* species within the family Pentatomidae. Comparative analysis using the previously sequenced mitogenomes of 14 Pentatomidae species revealed relatively low conservation in the control region, the length of which varied from 543 to 2,190 bp, and is the results of the number of repeating units copies. Furthermore, species invariably show different repeat unit even at the genus level. In contrast, we detected relatively limited variation with respect to the length of PCGs, tRNAs, and rRNAs, with PCGs showing the least variation (23 bp). Given their stable secondary structures, tRNAs and rRNAs also tend to contain conserved base pairs, such as A-G in the DHU stem and C-U in the anticodon stem (Hughes 1996).

We found that the A+T contents of Pentatomidae mitogenomes (72.05–77.43%) is significantly higher than that of G+C as other heteropteran species and is lower than the Lepidoptera mitogenomes (77.84–81.59%) (Hua et al. 2008, Wang et al. 2016, Li et al. 2017). Among the 13 PCGs, we found that the *atp8* and *cox1* genes have the highest and lowest A+T content, respectively, with content differing by more than 10%. Coincidentally, the *atp8* and *cox1* genes were found to have the highest (0.63) and lowest (0.05) Ka/Ks values, respectively. These results indicated that the evolutionary rate of proteins are related to their nucleotide composition, which is consistent with the view of Du et al. (2018). G+C content influences amino acid compositions, and then changes the proteins’ evolution (Jordan et al. 2005).

Most PCGs in heteropteran mitogenomes start with an ATN codon, although *cox1* begins with a TTG start codon (Hua et al. 2008, Yuan et al. 2015). Among Pentatomidae PCGs, TTG is the most frequently used start codon in *atp8* and *nad1*. Furthermore, among the synonymous codons encoding the same amino acid in Pentatomidae, those ending with an A or U are more frequently than those ending in a G or C, and also observed in other heteropteran species (Wang et al. 2015).

In general, a canonical clover-leaf secondary structure is predicted for tRNA genes; however, *trnSI* and *trnV* lack the DHU arm and form an aberrant loop structure (Johnson et al. 2018). Inaddition to the DHU arm, the TΨC stem also varies in length within different tRNAs (Navajas et al. 2002). Typically, the stem region structure of RNAs is more highly conserved than the loops, particularly the anticodon arm, and in line with expectations, we found that the anticodon arms in *trnW*, *trnK*, *trnL2* and *trnF* within Pentatomidae show 100% identity. For rRNAs, more than half of the sites are conserved within the Pentatomidae, with most of the conserved sites being located in stem regions.

The PCGs and PCGRNA databases used in the present study were evaluated for saturation and heterogeneity, and both were found to meet the criteria of phylogenetic analysis. In the ML and BI trees, the monophyly of different families within the Pentatomoidea were strongly supported based on the different datasets. The basal position of Urostylididae and the innermost position of Pentatomidae are consistent with previous hypotheses based on morphological data (Gapud 1991, Grazia et al. 2008) and molecular phylogenetic analyses (Grazia et al. 2008, Lis et al. 2012, Kocher et al. 2014). The Dinidoridae and Tessaratomidae formed a sister clade with nodal supports 100 for ML and 1.00 for BI. The close relationship of these two families was also suggested based on morphology (Henry 1997, Kment and Vilimova 2010) and molecular phylogenies (Grazia et al. 2008, Yuan et al. 2015).

The monophyly of tribes Strachiini and Pentatomini was consistently supported in ML and BI analyses with strong nodal support. The sister relationship of these two tribesis also suggested by morphological data (Yang 1962). In the past, the taxonomic status of the genus *Graphosoma* has tended to be somewhat ambiguous. On the basis of morphological analysis, most researchers adopt the view of that *Graphosoma* belongs to Podopinae (Rider 2006, Durak and Kalender 2009, Yuan et al. 2015). Nevertheless, many specialists hold the opinion that *Graphosoma* should be assigned to the tribe Graphosomini of Pentatominae. Although the phylogenetic analysis conducted in the present study was based a limited number of mitogenomes, our finding revealed that *G. rubrolineatum* is sister to Carpocorini, indicating that *Graphosoma* is a genus within the Pentatominae and not the Podopinae.

Our phylogenetic analyses also provided strong support for the assumed close relationship among *E. dominulus*, *E. gebleri*, and *E. maracandica*, and *E. ventralis*, *E. liturifure*, and *E. oleracea*, which were consistent with their morphological features. For instance, *E. dominulus*, *E. gebleri*, and *E. maracandica* were doubted to be identified due to unconspicuous morphological and structural differences, whereas abdominal spot variation in *E. ventralis*, *E. liturifera*, and *E. oleracea* is more obvious than that in the other three species.

In order to examine the contribution of different gene types, we assessed the PI of genes and partitions. We found that the PCGs are more variable than the RNA genes and that the phylogenetic information of the third position of the PCG codons is notably higher than that of either the first or second position. These results, therefore, indicated that the PCGs and particularly the third position of these genes constitute highly informative phylogenetic characters for the resolution of phylogenetic relationships (Nie et al. 2018).

In the present study, five mitogenomes from the Pentatomidae were added, the available mitogenomes for this group are still limited given the species richness. We accordingly emphasize the necessity for further sequencing of mitogenomes in the family Pentatomidae, which will contribute to the resolution of phylogenetic relationships.

## Supplementary Material

iez122_suppl_Supplementary_Material
